# Opportunities and barriers to translating the hibernation phenotype for neurocritical care

**DOI:** 10.3389/fneur.2023.1009718

**Published:** 2023-01-27

**Authors:** Kelly L. Drew, Saurav Bhowmick, Bernard W. Laughlin, Anna V. Goropashnaya, Øivind Tøien, M. Hoshi Sugiura, Ardy Wong, Kambiz Pourrezaei, Zeinab Barati, Chao-Yin Chen

**Affiliations:** ^1^Center for Transformative Research in Metabolism, Institute of Arctic Biology, University of Alaska Fairbanks, Fairbanks, AK, United States; ^2^Drexel University School of Biomedical Engineering, Science and Health Systems, Philadelphia, PA, United States; ^3^Barati Medical LLC, Fairbanks, AK, United States; ^4^Department of Pharmacology, University of California, Davis, Davis, CA, United States

**Keywords:** torpor, TTM, therapeutic hypothermia, ground squirrel, cerebral ischemia, ischemia/reperfusion, NIRS, neurocritical care

## Abstract

Targeted temperature management (TTM) is standard of care for neonatal hypoxic ischemic encephalopathy (HIE). Prevention of fever, not excluding cooling core body temperature to 33°C, is standard of care for brain injury post cardiac arrest. Although TTM is beneficial, HIE and cardiac arrest still carry significant risk of death and severe disability. Mammalian hibernation is a gold standard of neuroprotective metabolic suppression, that if better understood might make TTM more accessible, improve efficacy of TTM and identify adjunctive therapies to protect and regenerate neurons after hypoxic ischemia brain injury. Hibernating species tolerate cerebral ischemia/reperfusion better than humans and better than other models of cerebral ischemia tolerance. Such tolerance limits risk of transitions into and out of hibernation torpor and suggests that a barrier to translate hibernation torpor may be human vulnerability to these transitions. At the same time, understanding how hibernating mammals protect their brains is an opportunity to identify adjunctive therapies for TTM. Here we summarize what is known about the hemodynamics of hibernation and how the hibernating brain resists injury to identify opportunities to translate these mechanisms for neurocritical care.

## 1. Introduction

Cooling core body temperature (Tc) and hence brain temperature, termed targeted temperature management (TTM), remains standard of care for neonatal hypoxic-ischemic encephalopathy (HIE). For out of hospital cardiac arrest (OHCA) current guidelines recommend prevention of fever, not excluding cooling to 33°C. A focus on fever management vs. cooling stems from a challenge to demonstrate efficacy in large clinical trials (1). The struggle to demonstrate broad clinical efficacy of lowering Tc may be due to potential complications associated with shivering, disturbed hemodynamics, dysrhythmias and electrolyte disorders, particularly during the process of rewarming (2–4). Based on the magnitude of neuroprotection, metabolic suppression, lowered brain temperature and immune suppression, hibernation is a gold standard of neuroprotective adaptations (5–8).

### 1.1. Hibernation highlights the benefit of suppressing metabolism with a secondary fall in body temperature

Hibernation is an animal adaptation of energy conservation where a decrease in energy consumption precedes a fall in body temperature. For small (e.g., 0.3–1 kg) mammalian hibernators, evidence suggests that suppression of thermogenesis is sufficient to account for the initial fall in metabolic rate. A consequent decline in core body temperature suppresses metabolic rate further through thermodynamic influence on metabolic processes (9) as animals enter torpor, which we refer to here as hibernation torpor. The focus on suppressing thermogenesis to lower metabolic rate and produce a subsequent decrease in Tc distinguishes hibernation torpor from current TTM protocols to lower Tc or to prevent fever. If the physiology and neuroscience of hibernation was better understood, it could guide improved therapeutic strategies for TTM. Toward that end, research has revealed circuits to mimic fasting induced torpor in mice (10, 11). We have also found a necessary and sufficient role of central nervous system active, A_1_ adenosine receptor agonists to block thermogenesis and induce hibernation in ground squirrels (12). This mechanism, described as thermoregulatory inversion (13), can be mimicked in rats (14–16) and has inspired a new class of thermolytics designed to suppress thermogenesis within CNS thermoregulatory circuits (15). One example is a formulation of a centrally acting adenosine receptor agonist and a peripherally acting adenosine receptor antagonist (14, 17). This formulation is designed to target CNS A_1_ adenosine receptors to mimic natural hibernation with systemic drug administration (12, 16).

### 1.2. Hibernation illustrates integration of autonomic, thermoregulatory and metabolic processes

While new insights refine methods to suppress metabolism and lower body temperature, hibernation can also teach us about the integration of autonomic, thermoregulatory and metabolic processes needed to ensure that oxygen/nutrient supplies match the dramatic changes in metabolic load demonstrated in hibernation. Like with TTM, rewarming from hibernation may pose the greatest physiological challenge. One challenge is to match blood flow to metabolic load where metabolic rate increases from <2 percent of basal metabolic rate (BMR) during hibernation torpor to 300 percent of basal metabolic rate during the process of rewarming. Cellular adaptations underlying resistance to ischemia/reperfusion injury may have evolved as a necessary adaptation of heterothermy, to protect against ischemia/reperfusion injury during rewarming (5, 18–22). Indeed, the arctic ground squirrel (AGS) is known to resist ischemia/reperfusion injury in brain and other tissues (6, 23–25). Similarly, tightly regulated, and poorly understood, hemodynamics that guard against a mismatch between blood supply and demand may also have evolved to protect against ischemia/reperfusion injury during rewarming. Here we review what is known about hemodynamics and neuroprotection in hibernation and arousal from hibernation torpor and discuss what this can teach us about hemodynamic risk that will need to be mitigated for successful translation of synthetic torpor as a means to optimize the benefit of lowered Tc in humans.

## 2. Hemodynamics of hibernation

**Figure 1:**
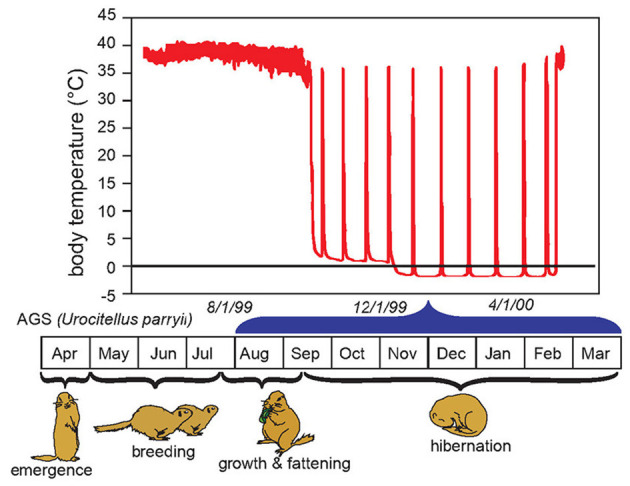
Body temperature in relation to life cycle and season of the hibernating arctic ground squirrel (AGS). The hibernation season, also referred to as hibernation, is marked by repeated bouts of prolonged torpor, termed here as hibernation torpor. Hibernation torpor is interrupted by spontaneous interbout arousals (IBA) noted by rapid increases in body temperature. Spontaneous IBAs are observed in all species of hibernators when core body temperature falls below 30°C (26). During arousal from hibernation, the nadir of mammalian metabolism is over-ridden by the high energetic costs of warming core body temperature from near 0°C to ~35°C in 2–3 h. During repeated recovery from hibernation oxygen consumption surges 300 fold from about 0.01 mLO_2_g^−1^h^−1^ to about 3 mLO_2_g^−1^h^−1^ (27). Arousal from torpor may be spontaneous or induced by external stimuli such as gentle handling. Once initiated, evidence suggests induced arousals proceed in the same way as natural arousals with the exception that induced arousals are faster and may be more energetically demanding [Adapted from Drew et al. (28)].

Hibernation torpor in AGS occurs during the winter season and consists of prolonged bouts of torpor lasting up to 3 weeks. These torpor bouts are interrupted by 12–24 h of interbout euthermia (Figure 1). Depending on ambient temperature during hibernation torpor body temperature may decrease to near or below 0°C, while the rate of oxygen consumption falls to 2% of BMR (29, 30). Hibernating hamsters show similar phenomena, but to slightly less extremes (31).

### 2.1. Heart rate declines with whole animal metabolic rate

Entrance into hibernation torpor is driven by metabolic suppression. Heart rate (HR) declines in synch with the decrease in metabolic rate to such a degree that HR is considered a proxy of metabolic rate (32). A defining hallmark of hibernation torpor is a hysteresis between oxygen consumption or HR and body temperature (33, 34) illustrating how a decrease in body temperature is secondary to a decrease in metabolic rate. Enhanced parasympathetic tone to the heart during entrance into hibernation torpor is evident from skipped heart beats (35, 36) which are reversed with atropine in hamsters (35). Onset of arousal is marked by withdrawal of parasympathetic and by an increase in sympathetic nervous system stimulation (35, 37). We know from these observations that the autonomic nervous system plays a fundamental role in regulating circulatory and thermoregulatory aspects of hibernation torpor (38), and potentially metabolic suppression.

### 2.2. Blood pressure closely tracks metabolic load

**Figure 2:**
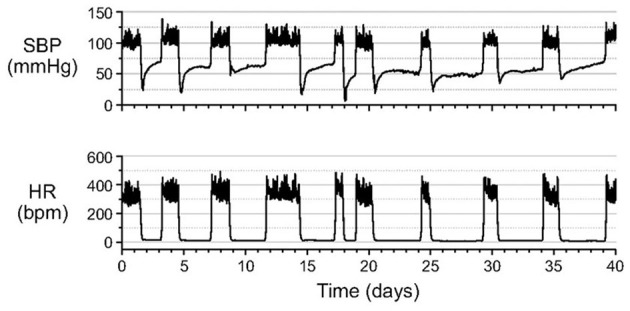
After entrance into hibernation torpor in Syrian hamsters, systolic blood pressure (SBP) increases to a new plateau at about 50% of euthermic systolic blood pressure (SBP). By contrast, heart rate (HR) remains at a steady minimum throughout the torpor bout. SBP and HR were measured in unanesthetized animals by telemetry with the catheter of a pressure transmitter inserted into the abdominal aorta [Horwitz et al. (33)].

As HR decreases during entrance into hibernation torpor so does blood pressure. A detailed study in hibernating hamsters illustrates that during entrance into hibernation torpor, systolic blood pressure (SBP) declines, presumably secondary to HR. However, while HR remains low and stable at <10% of euthermic HR throughout hibernation torpor, SBP increases to a new plateau at about 50% of euthermic SBP (Figure 2).

**Figure 3:**
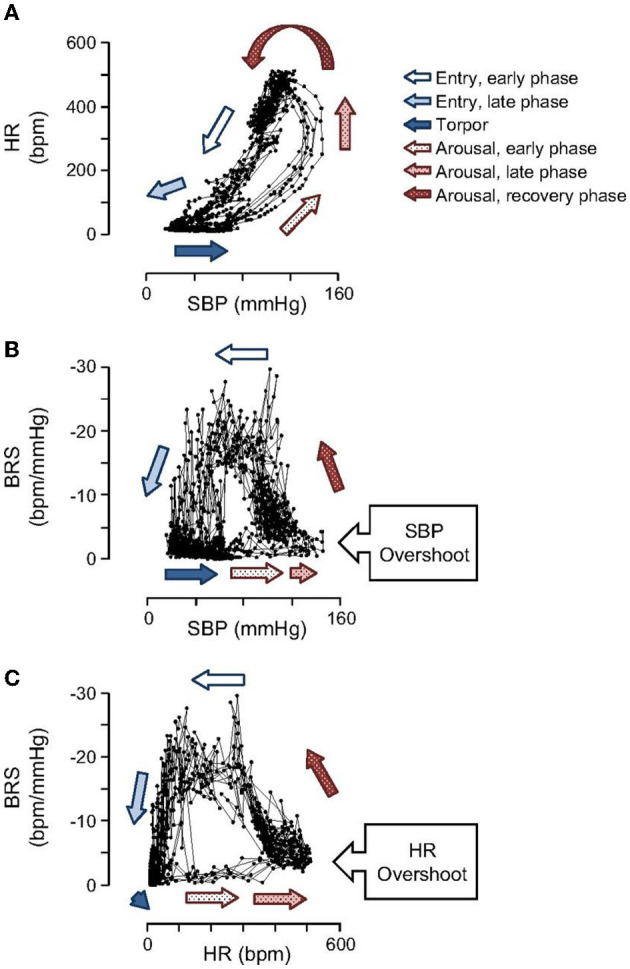
Open loops in hysteresis plots shown for SBP, HR and baroreceptor sensitivity (BRS) illustrate that cardiovascular control operates in fundamentally different ways during entrance and arousal. Importantly, the hysteresis between HR and SBP **(A)** illustrates that BP increases prior to HR during arousal and declines at the same rate as HR during entrance. During arousal, SBP **(B)** and HR **(C)** both increase to near maximal levels before BRS begins to increase [Horwitz et al. (33)].

During the early phase of arousal, blood pressure (BP) increases faster than HR, and during the late phase of arousal, HR increases significantly while BP stays at the highest level (Figure 3). BP shows a hysteresis relative to Tc that, except for a pronounced overshoot at the peak of arousal, resembles the hysteresis seen for HR and metabolic rate (33). Hysteresis between HR, metabolic rate and Tc illustrate how attenuated thermogenesis with a subsequent decrease in metabolic rate and HR during entrance precedes the decrease in Tc. The relationship between these variables differs during arousal where an unknown endogenous cue stimulates thermogenesis with a subsequent increase in metabolic rate and HR (9) and an eventual increase in Tc. During the early entrance phase the drop in BP follows the drop in HR in a fairly linear manner. As such, BP (perfusion pressure) closely tracks metabolic load or demand for oxygen. By tracking metabolic load, the animal can safely lower BP and hence oxygen supply while still matching the demand for oxygen.

### 2.3. Hibernation emphasizes the benefit of regulating blood pressure to meet metabolic load

Hibernation supports the idea that perfusion pressure must be optimized during TTM to meet metabolic load. During onset of hibernation torpor, BP decreases at the same rate as HR to match oxygen supply with oxygen demand. By contrast, at the onset of arousal, a steep increase in BP precedes an increase in HR. After BP reaches a maximum, HR continues to increase. The temporal relationship between HR and BP suggests that oxygen supply is increased in preparation for the increase in oxygen demand during rewarming and subsequent warm body temperature. This preemptive increase in oxygen supply is expected to contribute to successful rewarming. In humans, it may be beneficial to increase perfusion pressure before rewarming. In hibernation, the dramatic and rapid increase in BP that occurs before an increase in metabolic rate (inferred by an increase in HR), and before an increase in Tc (33) suggests that establishing adequate perfusion pressure before raising Tc may prevent brain injury during and after rewarming. This specific temporal relationship should guide rewarming procedures in development of synthetic torpor and potentially in current clinical application of TTM. Changes in baroreceptor sensitivity (BRS) during entrance and arousal demonstrates a functioning autonomic nervous system that modifies the BP setpoint as needed to optimize energy conservation without compromising brain and other vital tissue perfusion.

### 2.4. Dynamic modulation of baroreceptor sensitivity and vasoconstriction during hibernation optimizes perfusion pressure

Typically, as blood pressure declines, the baroreflex produces an increase in HR. Baroreceptor sensitivity (BRS) quantifies how much control the baroreflex has on the HR. Remarkably, BRS remains high during initial entrance into hibernation torpor, although HR continues to decrease despite a pronounced decrease in SBP. This temporal relationship suggests that BRS sensitivity is important during entrance into hibernation torpor. Moreover, it shows that the baroreflex is dynamically and effectively altering HR to ensure that BP tracks a declining set point during torpor entrance. More research is needed to define mechanisms that regulate the declining set point to understand how regulation could be optimized during TTM.

**Figure 4:**
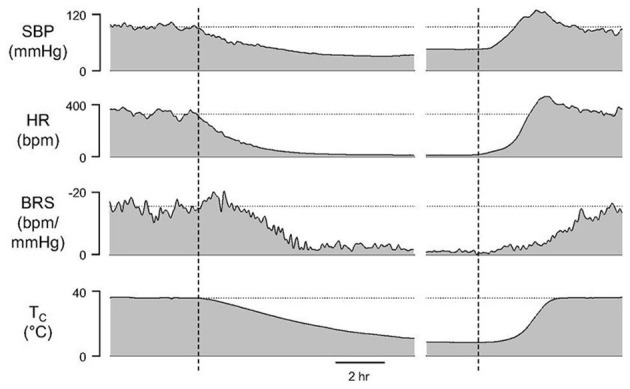
A gradual increase in HR is the first sign of arousal although a rapid increase in systolic blood pressure (SBP) precedes the subsequent, rapid rise in HR. Maximal SBP during arousal overshoots SBP measured during euthermia. Minimal baroreceptor sensitivity (BRS) at the onset of arousal may allow for the rapid rise in SBP and HR that are needed to support the metabolic demands of heart and brain as animals rewarm from hibernation torpor seen as a change in core body temperature (T_c_) [Horwitz et al. (33)].

BRS reaches a minimum at Tc of about 20°C and remains low throughout torpor and early arousal (Figure 4). Although a gradual increase in HR is the first sign of arousal, a rapid increase in SBP precedes the subsequent, rapid rise in HR and overshoots SBP measured during euthermia. Minimal BRS at the onset of arousal may allow for the rapid rise in BP and HR that are needed to support the metabolic demands of heart and brain as animals rewarm from hibernation torpor.

BP dynamics can also be explained in part by an increase in vasoconstriction and peripheral resistance throughout the hibernation season and during onset of arousal (33, 39, 40). Vasoconstriction is another feature of hibernation torpor that contributes to hemodynamics and distinguishes torpor from hypothermia. Increased vasoconstriction in ground squirrels (39, 41), hamsters and during fasting-induced torpor in mice (35, 41–43) decreases conductive heat loss. Peripheral resistance increases further at initiation of arousal, where touching an animal to induce arousal produces an immediate sympathetic surge to increase HR, metabolic rate, and vasoconstriction in the hindlimbs (40, 44). Peripheral vasoconstriction directs blood flow to the heart and brain. The classic pattern of rewarming in hibernating mammals begins with the thoracic area and brain and ends with the hindlimbs (44) with blood flow not returning fully to the hind limbs for as long as 48 h (45). Peripheral vasoconstriction combined with heat generated from brown fat creates pronounced heterogeneity of warming (38, 46).

### 2.5. Adaptations in hibernating species can guide the management of rewarming from TTM

What do these hemodynamic qualities mean for the brain and brain blood flow? During hibernation torpor cerebral blood flow, measured by quantitative autoradiography in 13 lined ground squirrels, falls from euthermic levels of 62 ± 18 mL 100 g^−1^ min^−1^ to an ischemic-like level of 7 ± 4 mL 100 g^−1^min^−1^ (47). By contrast, during arousal from hibernation torpor cerebral blood flow velocity peaks at 3.8 times the normal euthermic, resting levels (40, 44). Mathematical modeling of metabolic rate and parameters derived from ECG in hibernating 13 lined ground squirrel support the hypothesis that ground squirrels rewarm as quickly as is physiologically possible and that arousal from hibernation torpor is limited by capabilities of the cardiovascular system (48). Given human physiology, this rate of rewarming would not be possible in humans, however, it highlights adaptations in hibernating species that if better understood could guide the management of rewarming from TTM when Tc is lowered to 33°C.

## 3. Neuroprotection in hibernating species

Unique to hibernating species is an unprecedented resistance to cerebral ischemic/reperfusion injury, most likely necessary to tolerate interbout arousals illustrated in Figure 1. The innate neuroprotective phenotype of hibernating species must be appreciated to translate synthetic torpor to humans. While the essential biochemical or physiological processes filled by arousal episodes remain unknown, the significant energy reserves consumed by arousal (27, 49, 50) implies that they are needed for survival and thus hibernating mammals have evolved mechanisms to tolerate reperfusion of the heart and brain during times of peak oxidative metabolism. Arousal episodes challenge homeostasis in several ways and may be the most physiologically challenging aspect of heterothermy that if better understood could hold clues for TTM.

### 3.1. Despite optimized regulation of blood pressure, the brain of hibernating species resists ischemia reperfusion injury

**Figure 5:**
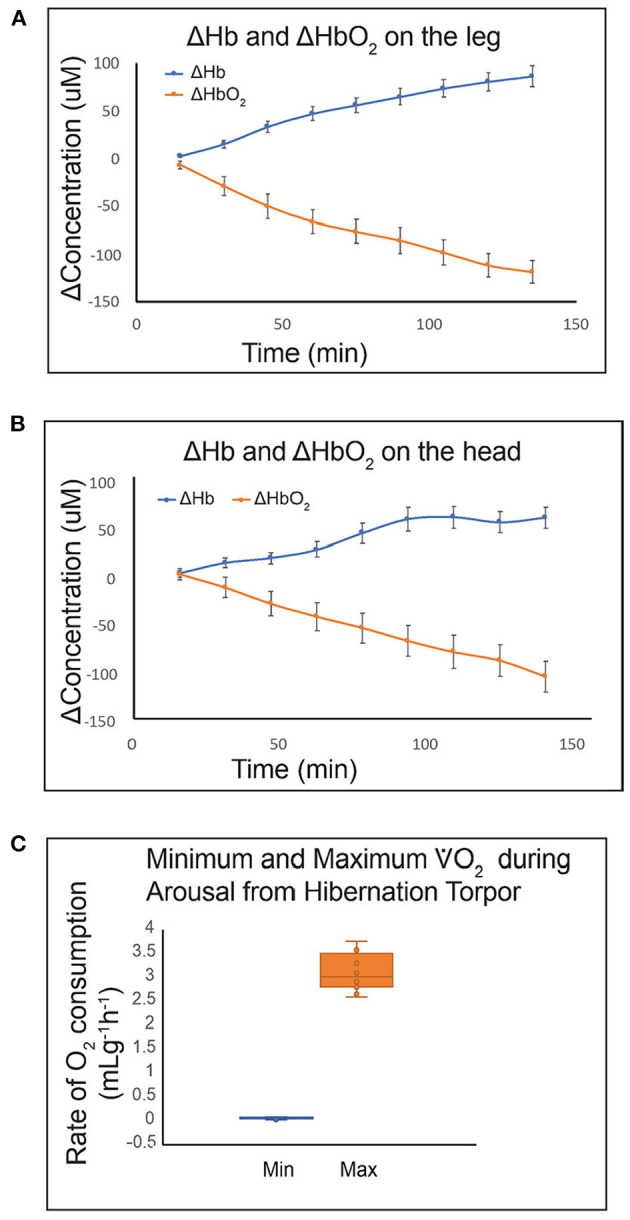
Use of a novel miniaturized near infrared spectroscopy (NIRS) device for quantifying Hb and HbO_2_ in small animals shows that HbO_2_ decreases during arousal from hibernation in hind leg **(A)** and brain **(B)** tissue. Hibernating AGS fit with sensors on the head and leg were placed in a metabolic cage at 0 min. Handling-induced arousal produced tissue hypoxia in both tissues. Rate of O_2_ consumption increased from 0.06 mLg^−1^h^−1^ at 0 min to a maximum of 3.1 mLg^−1^h^−1^ between 131 and 231 min. Data shown are mean ± SEM **(A, B)** and median with Q2 and Q3 defined by box and range shown as whiskers **(C)** (*n* = 8; 4M, 4F AGS, 10–11 months old).

**Figure 6:**
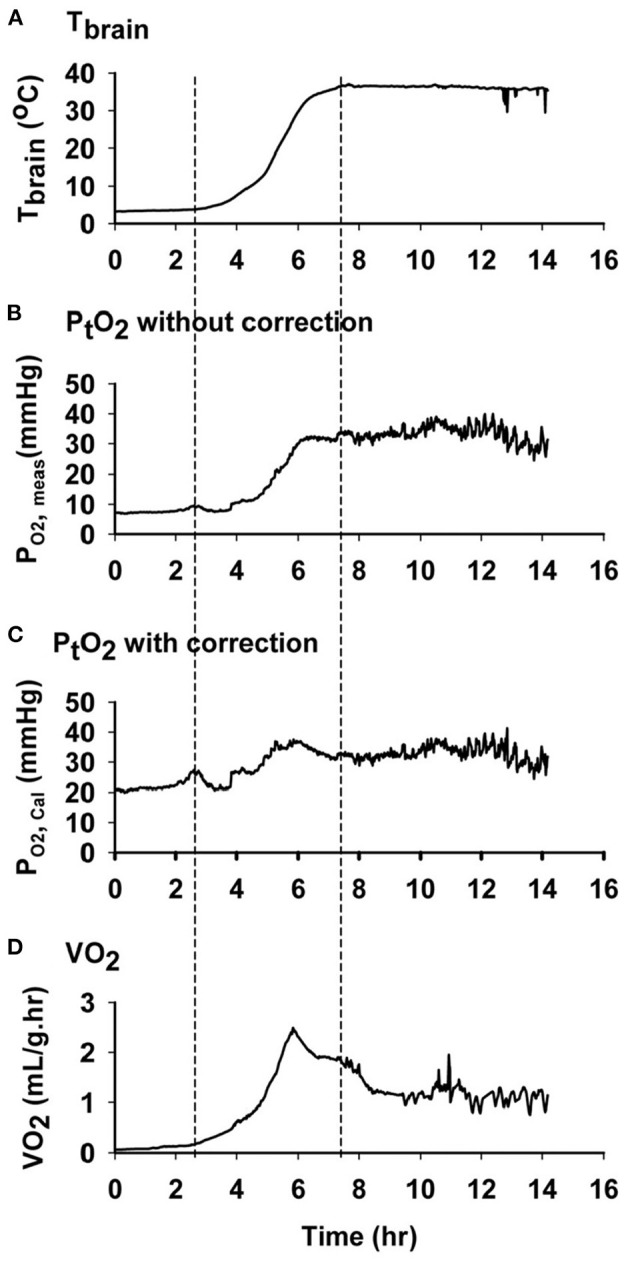
Oxygen concentration in AGS brain tissue does not decrease during arousal, despite a decrease in oxyhemoglobin. Data shown are representative graphs from a single AGS collected during an induced arousal from hibernation torpor at an ambient temperature of 2°C. First and second dashed line from left shows the time when arousal and euthermia started. **(A)** Changes in brain temperature during arousal. **(B)** Changes in PtO_2_ without temperature correction (P O_2_, meas). **(C)** Changes in calculated PtO_2_ with temperature correction (P O_2_, Cal). **(D)** Changes in the rate of oxygen consumption (VO_2_) measured by open flow respirometry [Ma and Wu (51) with permission].

The energy demanding process of interbout arousals puts animals at risk for ischemia/reperfusion if blood flow is not matched precisely with rising metabolic load as metabolic rate increases from two percent of BMR to over 300 percent of BMR within a few hours (27). For torpid hibernators housed near their thermoneutral zone (an ambient temperature of 0 to 2°C for an AGS), about 70% of energy reserves required for the entire hibernation season are consumed during arousal and subsequent episodes of euthermia (49, 50). During this period of high metabolic load in AGS, blood oxygen saturation (sO_2_) measured with a rectal, pulse-oximeter probe decreases to a minimum of 57% suggesting limited arterial blood oxygenation (22). Measure of brain tissue oxygenation using near infrared spectroscopy (NIRS) shows an increase in deoxyhemoglobin and a decrease in oxyhemoglobin in brain and hindleg during arousal (Figure 5). By contrast, direct measures of focal brain tissue oxygenation using an oxygen electrode implanted in striatum during arousal in AGS failed to show a significant decline in brain tissue O_2_ concentration (P_t_O_2_) (51) (Figure 6). One explanation why PtO_2_ may be preserved while oxyhemoglobin concentrations decline is the presence of an oxygen carrier or storage molecule such as neuroglobin.

### 3.2. An oxygen carrying molecule such as neuroglobin may add additional protection from a mismatch between perfusion pressure and metabolic load in hibernating species

**Figure 7:**
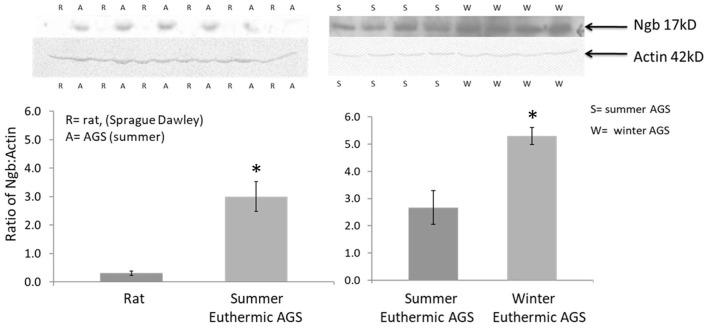
Western blots show higher expression of the 17kD neuroglobin monomer in cortex of AGS compared to cerebral ischemia sensitive rat and in cortex of euthermic AGS in winter compared to euthermic AGS in summer; 100 μg of protein was resolved on 10% SDS-PAGE and membranes were incubated with anti-Ngb (Ngb PolyAntibody (FL-151), 1:200, Santa Cruz Biotechnology, sc-30144) overnight followed by incubation with HRP-conjugated secondary antibody (Gt anti-rabbit IgG, 1:5,000, Santa Cruz Biotechnology). Optical density was normalized to actin. Rats were male, 3–4 months. AGS were male and female, adult (>1 year of age). ^*^P < 0.0003, *t*-test, *n* = 6 AGS vs rat; ^*^p < 0.0045, *t*-test, *n* = 4 summer vs. winter euthermic AGS [adapted from Bhowmick (52)].

Neuroglobin is a heme containing protein expressed in neurons. Preliminary data show that neuroglobin in brains of AGS is significantly higher than in rat brain (Figure 7). Neuroglobin is a member of the vertebrate globin family. Neuroglobin is best known for detoxifying NO and other reactive nitrogen species such as peroxinitrite (53). Neuroglobin may also serve as a storage and carrier molecule for O_2_. While binding affinity and other properties have failed to support such a role (54) the potential contribution of temperature during hibernation torpor and arousal on neuroglobin/O_2_ binding has not been studied. Low tissue temperature during torpor could load neuroglobin with O_2_. This O_2_ could then be released during rewarming upon arousal to maintain brain PtO_2_ despite falling tissue levels of oxyhemoglobin. How to improve O_2_ delivery to the penumbra in stroke is an active area of research with potential to enhance therapeutic efficacy of TTM (55, 56). Enhancing O_2_ delivery and titrating blood pressure to achieve perfusion/oxygenation targets show promise as a means to improve outcome after global cerebral ischemia (55, 57), but perfusion/oxygenation targets have yet to be optimized and routinely monitored during TTM (58) or during preclinical models of synthetic torpor.

### 3.3. Further neuroprotective measures protect the brain from potential mismatch between perfusion pressure and metabolic load

While the extent of or protection from cerebral hypoxia during arousal remains an area for further study, hemodynamic and neuroprotective measures appear to be optimized to minimize risk of ischemia/reperfusion injury when, during rewarming from hibernation torpor, cerebral blood flow returns from ischemic-like levels with an overshoot of SBP. The homeostatic challenges of interbout arousal may explain why ground squirrels have evolved to resist injury from ischemia reperfusion noted for several species and tissues (19–21, 23, 25, 59). Data suggests that temperature takes on an increasingly important protective role as tissue temperature declines (8, 22, 25). Thus, resistance to acute challenge as brain tissue warms during arousal is an important component of regulated transition out of hibernation torpor. Harnessing similar neuroprotective mechanisms for neurocritical care could synergize with therapeutic benefits of lowered brain temperature.

Even when not hibernating, AGS survive cardiac arrest with complete cessation of blood flow to the brain without evidence of neuropathology (6). AGS brain slices also tolerate prolonged periods of oxygen-glucose deprivation (OGD) *in vitro* (60) with no significant increase in neuronal cell death. Profound tolerance to cerebral ischemia, i.e., disruption in blood flow to the brain, is observed when AGS are not hibernating and when brain temperature is maintained near 37°C. Indeed, when the influence of temperature is excluded, cerebral ischemia tolerance in the euthermic state is so significant that it masks additional protection that may be afforded by hibernation torpor (20, 60, 61).

**Figure 8:**
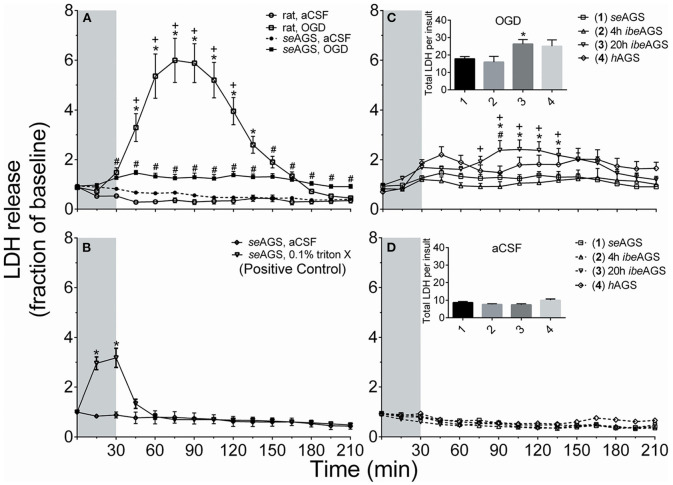
AGS brain tolerates OGD better than rat regardless of hibernation state or season. Cell death was measured from LDH released into the perfusion fluid in acute hippocampal slices from rat and euthermic AGS during the summer season (seAGS), during hibernation torpor (hAGS) and during early arousal (4 h ibeAGS) and late arousal (20 h ibeAGS), 4 and 20 h after initial handling to induce arousal. **(A)** LDH in perfusates increased in rat hippocampal slices exposed to OGD (rat, OGD), but not in rat slices exposed to artificial cerebral spinal fluid (aCSF) (rat, aCSF), nor in slices harvested from summer euthermic AGS and exposed to aCSF (seAGS, aCSF). A small amount of cell death is noted in slices collected from seAGS and exposed to OGD (seAGS, OGD). *p < 0.05 rat aCSF vs. rat OGD, ^+^*p* < 0.05 rat OGD vs. seAGS OGD, ^#^*p* < 0.05 seAGS aCSF vs. seAGS OGD. **(B)** As a positive control TritonX increased LDH release in seAGS slices (*p < 0.05 0.1% TritonX vs. aCSF). **(C)** AGS hippocampal slices are most vulnerable to OGD when collected from AGS 20 h into an interbout arousal (20 h ibeAGS). Insert shows the sum of LDH in perfusates collected 15–210 min from onset of OGD. *p < 0.05 seAGS vs. 20 h ibeAGS, ^+^*p* < 0.05 4 h ibeAGS vs. 20 h ibeAGS, ^#^*p* < 0.05 hAGS vs. 20 h ibeAGS, *t*-test with Bonferroni correction. **(D)** Exposure of slices from the same groups of animals as in **(C)** to aCSF has no effect on LDH release. Gray bar indicates 30 min treatment period. Data shown are means ± SEM, *n* = 4 slices in B, 25–30 slices per treatment in **(A, C, D)**. The novel microperfusion method, an improvement over previous use of propidium iodide as an indicator of cell death, replicated results obtained with propidium iodide [Bhowmick et al. (20)].

Figure 8 illustrates results from *in vitro* studies in acute AGS hippocampal slices. Using a novel microperfusion technique, we found that the innate neuroprotection of AGS persists at temperatures near 37°C regardless of hibernation season or state (20). These results replicate prior results (61). Data suggests that slices may be slightly vulnerable to OGD when harvested from animals after the peak in metabolic load, during the final stage of an interbout arousal. However, even at this vulnerable period AGS hippocampal slices resist injury significantly better than slices from Sprague Dawley rat. Resistance to OGD injury persists despite a loss of ATP (20, 61), delayed, but eventual depolarization (5), and release of glutamate and acidosis (19, 20). Downstream to these events AGS tend to buffer intracellular calcium better than rat (62) and mitigate oxidative and nitrosative stress; in particular AGS brain resists peroxynitrite-mediated injury (19). A working hypothesis that warrants further study is that the high expression of neuroglobin in AGS brain aides in resistance to peroxynitrite-mediated injury.

### 3.4. Neuroprotective adaptations may be complimented by regenerative processes stimulated by mild ischemia/reperfusion

Other data suggests that enhanced capacity for neural progenitor survival and proliferation contributes to neuroprotection in AGS. Oxygen glucose deprivation or hypoxia alone, promote proliferation of AGS neural progenitor cells isolated from AGS hippocampus (63). In an elegant study of these cells, Singhal et al. (18), found that a natural AGS variant of the mitochondrial protein ATP5G1 contributes to resistance to metabolic stress. Specifically, Leu 32 in the AGS ATP5G1 protein enhances respiratory capacity and preserves mitochondrial morphology when cells are exposed to hypoxic challenge. Singhal's study also identified mesencephalic astrocyte-derived neurotrophic factor and calmodulin as cytoprotective proteins in AGS neural progenitor cells (18).

Observations *in vivo* complement evidence *in vitro* that arousal from hibernation promotes regenerative processes. Seasonal hibernators do not suffer cognitive deficits after interbout arousal. Although some forms of learning acquired prior to the onset of the hibernation season are compromised after final arousal in spring (64), we and others have reported evidence of enhanced cognitive capacities after interbout arousal (65, 66). Arousal from torpor in mice also enhances synaptic strength and improves memory in a mouse model of Alzheimer's disease (67). Hibernation and cooling in non-hibernating species increase expression of cold-shock, RNA binding proteins such as RBM3 which promotes synaptogenesis (68–70) and skeletal muscle hypertrophy (71). These lessons point to an opportunity to study the benefit of regenerative therapies post TTM.

### 3.5. Hibernation argues for complementary neuroprotective and regenerative adjunctive therapies to enhance the efficacy of targeted temperature management

HIE treated with TTM of 33°C still leads to severe complications with 48 percent of cases ending in death or moderate to severe disability (72). Adjunctive therapies for TTM are in development for HIE (73). Of the approximately 350,000 people who suffer an OHCA and are treated by EMS, only 6 to 16 percent survive (74) despite access to TTM. Clearly there is a need to optimize benefit of TTM, potentially by turning the focus from temperature to metabolic suppression and cerebral oxygenation. There is also an opportunity to add neuroprotective and regenerative therapies to TTM. Since the HACA and Bernard trials in 2002 the frequency of cooling to 33°C post OHCA grew with advances of cooling devices. During this time the proportion of patients with ROSC after OHCA who survive to hospital discharge also increased (75). Nonetheless, the more recent, large, well-controlled and well-designed TTM2 trial found no benefit of lowering core body temperature to 33°C over fever management (76), but the neuroprotective benefit of hibernation torpor (8) emphasizes the benefit of lowered brain temperature. Hibernation also suggests that lowered temperature should be secondary to the inhibition of thermogenesis and metabolic suppression. Without metabolic suppression and a suppressed cold-defense response the efficacy of cooling *per-se* will likely remain variable and limited. Similarly, bradycardia and hypotension are consequences of cooling and may or may not be sufficient to sustain sufficient cerebral perfusion pressure. Sufficiency of BP during lowered Tc could be determined if brain tissue oxygenation was monitored. By contrast, hypotension during rewarming may counteract the benefit of cooling by failing to meet the metabolic demands of a warming brain. To translate synthetic torpor to the clinic, cerebral oxygenation targets during TTM and rewarming should be followed. Other neuroprotective and regenerative adaptations in hibernating animals should also be investigated and developed as adjunctive therapies for TTM.

## Author contributions

All authors listed have made a substantial, direct, and intellectual contribution to the work and approved it for publication.

## References

[1] Dankiewicz J Cronberg T Lilja G Jakobsen JC Levin H Ullen S. Hypothermia versus normothermia after out-of-hospital cardiac arrest. N Engl J Med. (2021) 384:2283–94. 10.1056/NEJMoa2100591 34133859

[2] Bro-Jeppesen J Annborn M Hassager C Wise MP Pelosi P Nielsen N. Hemodynamics and vasopressor support during targeted temperature management at 33 degrees c versus 36 degrees c after out-of-hospital cardiac arrest: a post hoc study of the target temperature management trial^*^. Crit Care Med. (2015) 43:318–27. 10.1097/CCM.0000000000000691 25365723

[3] Weant KA Martin JE Humphries RL Cook AM. Pharmacologic options for reducing the shivering response to therapeutic hypothermia. Pharmacotherapy. (2010) 30:830–41. 10.1592/phco.30.8.830 20653360

[4] Polderman KH. Mechanisms of action, physiological effects, and complications of hypothermia. Crit Care Med. (2009) 37:S186–202. 10.1097/CCM.0b013e3181aa5241 19535947

[5] Dave KR Anthony Defazio R Raval AP Dashkin O Saul I Iceman KE. Protein kinase c epsilon activation delays neuronal depolarization during cardiac arrest in the euthermic arctic ground squirrel. J Neurochem. (2009) 110:1170–9. 10.1111/j.1471-4159.2009.06196.x 19493168 PMC2774829

[6] Dave KR Prado R Raval AP Drew KL Perez-Pinzon MA. The arctic ground squirrel brain is resistant to injury from cardiac arrest during euthermia. Stroke. (2006) 37:1261–5. 10.1161/01.STR.0000217409.60731.38 16574920

[7] Drew KL Rice ME Kuhn TB Smith MA. Neuroprotective adaptations in hibernation: Therapeutic implications for ischemia-reperfusion, traumatic brain injury and neurodegenerative diseases. Free Radic Biol Med. (2001) 31:563–73. 10.1016/S0891-5849(01)00628-1 11522441

[8] Zhou F Zhu XW Castellani RJ Stimmelmayr R Perry G Smith MA. Hibernation, a model of neuroprotection. Am J Pathol. (2001) 158:2145–51. 10.1016/S0002-9440(10)64686-X 11395392 PMC1891987

[9] Geiser F. Ecological Physiology of Daily Torpor and Hibernation. Berlin, Germany: Springer. (2021). pp. 133–6. 10.1007/978-3-030-75525-6

[10] Hrvatin S Sun S Wilcox OF Yao H Lavin-Peter AJ Cicconet M. Neurons that regulate mouse torpor. Nature. (2020) 583:115–21. 10.1038/s41586-020-2387-5 32528180 PMC7449701

[11] Takahashi TM Sunagawa GA Soya S Abe M Sakurai K Ishikawa K. A discrete neuronal circuit induces a hibernation-like state in rodents. Nature. (2020) 583:109–14. 10.1038/s41586-020-2163-6 32528181

[12] Jinka TR Tøien Ø Drew KL. Season primes the brain in an arctic hibernator to facilitate entrance into torpor mediated by adenosine a(1) receptors. J Neurosci. (2011) 31:10752–8, 3325781. 10.1523/JNEUROSCI.1240-11.2011 21795527 PMC3325781

[13] Tupone D Cano G Morrison SF. Thermoregulatory inversion: a novel thermoregulatory paradigm. Am J Physiol Regul Integr Comp Physiol. (2017) 312:R779–86. 10.1152/ajpregu.00022.2017 28330964 PMC5451569

[14] Laughlin BW Bailey IR Rice SA Barati Z Bogren LK Drew KL. Precise control of target temperature using n(6)-cyclohexyladenosine and real-time control of surface temperature. Ther Hypothermia Temp Manag. (2018) 8:108–16. 10.1089/ther.2017.0020 29480748 PMC5994145

[15] Cerri M Mastrotto M Tupone D Martelli D Luppi M Perez E. The inhibition of neurons in the central nervous pathways for thermoregulatory cold defense induces a suspended animation state in the rat. J Neurosci. (2013) 33:2984–93. 10.1523/JNEUROSCI.3596-12.2013 23407956 PMC6619194

[16] Tupone D Madden CJ Morrison SF. Central activation of the a1 adenosine receptor (a1ar) induces a hypothermic, torpor-like state in the rat. J Neurosci. (2013) 33:14512–25. 10.1523/JNEUROSCI.1980-13.2013 24005302 PMC3761054

[17] Jinka TR Combs VM Drew KL. Translating drug-induced hibernation to therapeutic hypothermia. ACS Chem Neurosci. (2015) 6:899–904. 10.1021/acschemneuro.5b00056 25812681 PMC4939144

[18] Singhal NS Bai M Lee EM Luo S Cook KR Ma DK. Cytoprotection by a naturally occurring variant of atp5g1 in arctic ground squirrel neural progenitor cells. Elife. (2020) 9:e55578. 10.7554/eLife.55578 33050999 PMC7671683

[19] Bhowmick S Drew KL. Arctic ground squirrel resist peroxynitrite-mediated cell death in response to oxygen glucose deprivation. Free Radic Biol Med. (2017) 113:203–11. 10.1016/j.freeradbiomed.2017.09.024 28962873 PMC5699938

[20] Bhowmick S Moore JT Kirschner DL Drew KL. Arctic ground squirrel hippocampus tolerates oxygen glucose deprivation independent of hibernation season even when not hibernating and after atp depletion, acidosis, and glutamate efflux. J Neurochem. (2017) 142:160–70. 10.1111/jnc.13996 28222226 PMC5479730

[21] Lee YJ Miyake S Wakita H McMullen DC Azuma Y Auh S. Protein sumoylation is massively increased in hibernation torpor and is critical for the cytoprotection provided by ischemic preconditioning and hypothermia in shsy5y cells. J Cereb Blood Flow Metab. (2007) 27:950–62. 10.1038/sj.jcbfm.9600395 16955077 PMC2396349

[22] Ma YL Zhu X Rivera PM Tøien Ø Barnes BM LaManna JC. Absence of cellular stress in brain after hypoxia induced by arousal from hibernation in arctic ground squirrels. Am J Physiol Regul Integr Comp Physiol. (2005) 289:R1297–1306. 10.1152/ajpregu.00260.2005 15976308

[23] Bogren LK Olson JM Carpluk J Moore JM Drew KL. Resistance to systemic inflammation and multi organ damage after global ischemia/reperfusion in the arctic ground squirrel. PLoS ONE. (2014) 9:e94225, 3984146. 10.1371/journal.pone.0094225 24728042 PMC3984146

[24] Kurtz CC Lindell SL Mangino MJ Carey HV. Hibernation confers resistance to intestinal ischemia-reperfusion injury. Am J Physiol Gastrointest Liver Physiol. (2006) 291:G895–901. 10.1152/ajpgi.00155.2006 16751173

[25] Frerichs KU Hallenbeck JM. Hibernation in ground squirrels induces state and species-specific tolerance to hypoxia and aglycemia: An *in vitro* study in hippocampal slices. J Cereb Blood Flow Metab. (1998) 18:168–75. 10.1097/00004647-199802000-00007 9469159

[26] Dausmann KH Glos J Ganzhorn JU Heldmaier G. Physiology: Hibernation in a tropical primate. Nature. (2004) 429:825–6. 10.1038/429825a 15215852

[27] Tøien Ø Drew KL Chao ML Rice ME. Ascorbate dynamics and oxygen consumption during arousal from hibernation in arctic ground squirrels. Am J Physiol Regul Integr Comp Physiol. (2001) 281:R572–583. 10.1152/ajpregu.2001.281.2.R572 11448862

[28] Drew KL Harris MB LaManna JC Smith MA Zhu XW Ma YL. Hypoxia tolerance in mammalian heterotherms. J Exp Biol. (2004) 207:3155–62. 10.1242/jeb.01114 15299037

[29] Buck CL Barnes BM. Effects of ambient temperature on metabolic rate, respiratory quotient, and torpor in an arctic hibernator. Am J Physiol Regul Integr Comp Physiol. (2000) 279:R255–262. 10.1152/ajpregu.2000.279.1.R255 10896889

[30] Barnes BM. Freeze avoidance in a mammal: Body temperatures below 0 degree c in an arctic hibernator. Science. (1989) 244:1593–5. 10.1126/science.2740905 2740905

[31] Geiser F. Metabolic rate and body temperature reduction during hibernation and daily torpor. Annu Rev Physiol. (2004) 66:239–74. 10.1146/annurev.physiol.66.032102.115105 14977403

[32] Currie SE Kortner G Geiser F. Heart rate as a predictor of metabolic rate in heterothermic bats. J Exp Biol. (2014) 217:1519–24. 10.1242/jeb.098970 24436390

[33] Horwitz BA Chau SM Hamilton JS Song C Gorgone J Saenz M. Temporal relationships of blood pressure, heart rate, baroreflex function, and body temperature change over a hibernation bout in syrian hamsters. Am J Physiol Regul Integr Comp Physiol. (2013) 305:R759–768. 10.1152/ajpregu.00450.2012 23904107 PMC3798792

[34] Mertens A Stiedl O Steinlechner S Meyer M. Cardiac dynamics during daily torpor in the djungarian hamster (phodopus sungorus). Am J Physiol Regul Integr Comp Physiol. (2008) 294:R639–650. 10.1152/ajpregu.00496.2007 18032471

[35] Lyman CP O'Brien RC. Autonomic control of circulation during the hibernating cycle in ground squirrels. J Physiol. (1963) 168:477–99. 10.1113/jphysiol.1963.sp007204 14067940 PMC1359436

[36] Lyman CP. Oxygen consumption, body temperature and heart rate of woodchucks entering hibernation. Am J Physiol. (1958) 194:83–91. 10.1152/ajplegacy.1958.194.1.83 13559433

[37] Zanetti F Chen CY Baker HA Sugiura MH Drew KL Barati ZB. Cardiac rhythms and variation in hibernating arctic ground squirrels. Physiol Biochem Zool. (In Press). 10.1086/724688 PMC12228421 37278587

[38] Drew KL. Raising the 'dead' - reperfusion from torpor. J Exp Biol. (2013) 216:927–9. 10.1242/jeb.076174 23447661

[39] Frare C Jenkins ME McClure KM Drew KL. Seasonal decrease in thermogenesis and increase in vasoconstriction explain seasonal response to n(6) -cyclohexyladenosine-induced hibernation in the arctic ground squirrel (urocitellus parryii). J Neurochem. (2019) 151:316–35. 10.1111/jnc.14814 31273780 PMC6819227

[40] Osborne PG Sato J Shuke N Hashimoto M. Sympathetic alpha-adrenergic regulation of blood flow and volume in hamsters arousing from hibernation. Am J Physiol Regul Integr Comp Physiol. (2005) 289:R554–62. 10.1152/ajpregu.00004.2005 15845885

[41] Karoon P Knight G Burnstock G. Enhanced vasoconstrictor responses in renal and femoral arteries of the golden hamster during hibernation. J Physiol. (1998) 512 (Pt 3):927–38. 10.1111/j.1469-7793.1998.927bd.x 9769433 PMC2231249

[42] Swoap SJ Gutilla MJ. Cardiovascular changes during daily torpor in the laboratory mouse. Am J Physiol Regul Integr Comp Physiol. (2009) 297:R769–774. 10.1152/ajpregu.00131.2009 19587115 PMC3774276

[43] Lyman L O'Brien R. Circulatory changes in the thirteen-lined ground squirrel during the hibernation cycle. Mammalian Hibernation. Bull Mus Comp Zool. (1960) 124:353–72.

[44] Osborne PG Hashimoto M. State-dependent regulation of cortical blood flow and respiration in hamsters: Response to hypercapnia during arousal from hibernation. J Physiol. (2003) 547:963–70. 10.1113/jphysiol.2002.033571 12576499 PMC2342736

[45] Drew KL Zuckerman JA Shenk PE Bogren LK Jinka TR Moore JT. Hibernation: a natural model of tolerance to cerebral ischemia/reperfusion. In:Gidday J Perez-Pinzon M, editors. Innate Neuroprotection for Stroke. New York: Springer. (2012). p. 37–50.23775726

[46] Ballinger MA Andrews MT. Nature's fat-burning machine: Brown adipose tissue in a hibernating mammal. J Exp Biol. (2018) 221:jeb162586. 10.1242/jeb.162586 29514878 PMC6919643

[47] Frerichs KU Kennedy C Sokoloff L Hallenbeck JM. Local cerebral blood flow during hibernation, a model of natural tolerance to “cerebral ischemia”. J Cereb Blood Flow Metab. (1994) 14:193–205. 10.1038/jcbfm.1994.26 8113316

[48] Hampton M Andrews MT. A simple molecular mathematical model of mammalian hibernation. J Theor Biol. (2007) 247:297–302. 10.1016/j.jtbi.2007.03.005 17459419 PMC2580757

[49] Karpovich SA Tøien Ø Buck CL Barnes BM. Energetics of arousal episodes in hibernating arctic ground squirrels. J Comp Physiol B. (2009) 179:691–700. 10.1007/s00360-009-0350-8 19277682

[50] Heldmaier G Ortmann S Elvert R. Natural hypometabolism during hibernation and daily torpor in mammals. Respir Physiol Neurobiol. (2004) 141:317–29. 10.1016/j.resp.2004.03.014 15288602

[51] Ma Y Wu S. Simultaneous measurement of brain tissue oxygen partial pressure, temperature, and global oxygen consumption during hibernation, arousal, and euthermy in non-sedated and non-anesthetized arctic ground squirrels. J Neurosci Methods. (2008) 174:237–44, 2615241. 10.1016/j.jneumeth.2008.07.011 18722471 PMC2615241

[52] Bhowmick S. Modulation of Ischemia- Reperfusion Injury in Mammalian Hibernators and Non-hibernators: A Comparative Study. (2017).

[53] Herold S Fago A. Reactions of peroxynitrite with globin proteins and their possible physiological role. Comp Biochem Physiol A Mol Integr Physiol. (2005) 142:124–9. 10.1016/j.cbpb.2005.06.009 16055362

[54] Fago A Hundahl C Dewilde S Gilany K Moens L Weber RE. Allosteric regulation and temperature dependence of oxygen binding in human neuroglobin and cytoglobin. Molecular mechanisms and physiological significance. J Biol Chem. (2004) 279:44417–26. 10.1074/jbc.M407126200 15299006

[55] Deuchar GA van Kralingen JC Work LM Santosh C Muir KW McCabe C. Preclinical validation of the therapeutic potential of glasgow oxygen level dependent (gold) technology: a theranostic for acute stroke. Transl Stroke Res. (2019) 10:583–95. 10.1007/s12975-018-0679-y 30506268 PMC6733820

[56] Baron JC. Protecting the ischaemic penumbra as an adjunct to thrombectomy for acute stroke. Nat Rev Neurol. (2018) 14:325–37. 10.1038/s41582-018-0002-2 29674752

[57] Elmer J Flickinger KL Anderson MW Koller AC Sundermann ML Dezfulian C. Effect of neuromonitor-guided titrated care on brain tissue hypoxia after opioid overdose cardiac arrest. Resuscitation. (2018) 129:121–6. 10.1016/j.resuscitation.2018.04.013 29679696 PMC6054552

[58] Hosseini M Wilson RH Crouzet C Amirhekmat A Wei KS Akbari Y. Resuscitating the globally ischemic brain: Ttm and beyond. Neurotherapeutics. (2020) 17:539–62. 10.1007/s13311-020-00856-z 32367476 PMC7283450

[59] Lindell SL Klahn SL Piazza TM Mangino MJ Torrealba JR Southard JH. Natural resistance to liver cold ischemia-reperfusion injury associated with the hibernation phenotype. Am J Physiol Gastrointest Liver Physiol. (2005) 288:G473–480. 10.1152/ajpgi.00223.2004 15701622

[60] Ross AP Christian SL Zhao HW Drew KL. Persistent tolerance to oxygen and nutrient deprivation and n-methyl-d-aspartate in cultured hippocampal slices from hibernating arctic ground squirrel. J Cereb Blood Flow Metab. (2006) 26:1148–56. 10.1038/sj.jcbfm.9600271 16395285

[61] Christian SL Ross AP Zhao HW Kristenson HJ Zhan X Rasley BT. Arctic ground squirrel (*spermophilus parryii*) hippocampal neurons tolerate prolonged oxygen-glucose deprivation and maintain baseline erk1/2 and jnk activation despite drastic atp loss. J Cereb Blood Flow Metab. (2008) 28:1307–19. 10.1038/jcbfm.2008.20 18398417 PMC2792705

[62] Zhao HW Ross AP Christian SL Buchholz JN Drew KL. Decreased nr1 phosphorylation and decreased nmdar function in hibernating arctic ground squirrels. J Neurosci Res. (2006) 84:291–8. 10.1002/jnr.20893 16676330 PMC3796386

[63] Drew KL Wells M McGee R Ross AP Kelleher-Andersson J. Arctic ground squirrel neuronal progenitor cells resist oxygen and glucose deprivation-induced death. World J Biol Chem. (2016) 7:168–77. 10.4331/wjbc.v7.i1.168 26981205 PMC4768121

[64] Millesi E Prossinger H Dittami JP Fieder M. Hibernation effects on memory in european ground squirrels (spermophilus citellus). J Biol Rhythms. (2001) 16:264–71. 10.1177/074873001129001971 11407786

[65] Weltzin MM Zhao HW Drew KL Bucci DJ. Arousal from hibernation alters contextual learning and memory. Behav Brain Res. (2006) 167:128–33. 10.1016/j.bbr.2005.08.021 16219369 PMC12186774

[66] Mihailovic L Petrovic-Minic B Protic S Divac I. Effects of hibernation on learning and retention. Nature. (1968) 218:191–2. 10.1038/218191a0 5645294

[67] de Veij Mestdagh CF Timmerman JA Koopmans F Paliukhovich I Miedema SSM Goris M. Torpor enhances synaptic strength and restores memory performance in a mouse model of alzheimer's disease. Sci Rep. (2021) 11:15486. 10.1038/s41598-021-94992-x 34326412 PMC8322095

[68] Jackson TC Kochanek PM. A new vision for therapeutic hypothermia in the era of targeted temperature management: a speculative synthesis. Ther Hypothermia Temp Manag. (2019) 9:13–47. 10.1089/ther.2019.0001 30802174 PMC6434603

[69] Peretti D Bastide A Radford H Verity N Molloy C Martin MG. Rbm3 mediates structural plasticity and protective effects of cooling in neurodegeneration. Nature. (2015) 518:236–9. 10.1038/nature14142 25607368 PMC4338605

[70] Fedorov VB Goropashnaya AV Tøien Ø Stewart NC Gracey AY Chang C. Elevated expression of protein biosynthesis genes in liver and muscle of hibernating black bears (ursus americanus). Physiol Genomics. (2009) 37:108–18. 10.1152/physiolgenomics.90398.2008 19240299 PMC3774579

[71] Van Pelt DW Confides AL Judge AR Vanderklish PW Dupont-Versteegden EE. Cold shock protein rbm3 attenuates atrophy and induces hypertrophy in skeletal muscle. J Muscle Res Cell Motil. (2018) 39:35–40. 10.1007/s10974-018-9496-x 30051360

[72] Tagin MA Woolcott CG Vincer MJ Whyte RK Stinson DA. Hypothermia for neonatal hypoxic ischemic encephalopathy: an updated systematic review and meta-analysis. Arch Pediatr Adolesc Med. (2012) 166:558–66. 10.1001/archpediatrics.2011.1772 22312166

[73] Victor S Rocha-Ferreira E Rahim A Hagberg H Edwards D. New possibilities for neuroprotection in neonatal hypoxic-ischemic encephalopathy. Eur J Pediatr. (2022) 181:875–87. 10.1007/s00431-021-04320-8 34820702 PMC8897336

[74] Virani SS Alonso A Aparicio HJ Benjamin EJ Bittencourt MS Callaway CW. Heart disease and stroke statistics-2021 update: a report from the american heart association. Circulation. (2021) 143:e254–743. 33501848 10.1161/CIR.0000000000000950 PMC13036842

[75] Bonaventura J Alan D Vejvoda J Honek J Veselka J. History and current use of mild therapeutic hypothermia after cardiac arrest. Arch Med Sci. (2016) 12:1135–41. 10.5114/aoms.2016.61917 27695505 PMC5016592

[76] Nielsen N Wetterslev J Cronberg T Erlinge D Gasche Y Hassager C. Targeted temperature management at 33 degrees c versus 36 degrees c after cardiac arrest. N Engl J Med. (2013) 369:2197–206. 10.1056/NEJMoa1310519 24237006

